# Supplementary material for: Comparative Gene-Expression Analysis of Alzheimer’s Disease Progression with Aging in Transgenic Mouse Model
Source: Int J Mol Sci. 2019 Mar 11;20(5):1219. doi: 10.3390/ijms20051219 (PMC6429175; doi:10.3390/ijms20051219)
Supplement: Supplementary file 1 [file ijms-20-01219-s001.pdf]

GENES	6 MONTH		9 MONTH		12 MONTH	
	Over expression	Under expression	Over expression	Under expression	Over expression	Under expression
ABAD	●		●		●	
ADAM10		●		●		●
APH1B		●		●		●
APOE		●		●		●
APP	●		●		●	
APPBP1	●		●		●	
ATF6		●		●		●
BACE1				●		●
BAD			●			
BID			●		●	
CALM1		●		●		●
CALN	●		●		●	
CASP12		●			●	
CASP3	●		●			
CASP7	●		●		●	
CASP8	●		●		●	
CASP9	●		●		●	
CDK5	●		●			●
CXI						
CXII				●		
CXIII	●				●	
CXV				●		●
CXVI		●		●		●
CYCS	●		●		●	
ERK/MAPK2		●	●		●	
FAS	●		●			
GQ				●		●
GSK3B	●		●		●	
IDE		●	●		●	
IL1B		●	●		●	
IP3R	●		●		●	
IRE1A	●		●		●	
LPL			●		●	
LRP1	●			●		●
MAPT/TAU	●		●		●	
NEP/MME		●		●		●
NMDAR	●		●		●	
NOS1		●	●		●	
P25					●	

PEN-2			●			
PERK				●	●	●
PLCB1			●		●	
PSEN1				●		●
RYR3	●		●		●	
SERCA1	●					●
SNCA 1		●		●		●
TNF	●		●		●	
VDCC		●	●		●	

Table S 1: Genes up regulated or down regulated with age progression

Gene Symbol	Function	Description
ADAM10	Signal transduction , Metallopeptidase.	A disintegrin and metallopeptidase domain 10
ABAD	Metabolism (Mitochondia) Amino acid metabolism.	3-hydroxyacyl-CoA dehydrogenase / 3-hydroxy-2-methylbutyryl-CoA dehydrogenase
APH1B	Environmental Information Processing Signal transduction.	anterior pharynx defective 1
Apo-E	Exosomal protein.	apolipoprotein E
App	Serotonergic synapse	amyloid beta A4 protein
Atf6	Protein processing in endoplasmic reticulum	cyclic AMP-dependent transcription factor ATF-6 alpha (ER procesing)
Serca1	Environmental Information Processing Signal transduction ,Calcium signaling pathway	ATPase, Ca++ transporting, cardiac muscle, fast twitch 1 .Ca2+ transporting ATPase, sarcoplasmic/endoplasmic reticulum
Bace1	Hydrolase, Acting on peptide bonds	beta-site APP-cleaving enzyme 1 .
Bad	Signal transduction Ras,ErbB, VEGF ,cAMP cGMP - PKG ,PI3K-Akt signaling pathway.	BCL2-associated agonist of cell death
Bid	Signal transduction, Sphingolipid signaling pathway	BH3 interacting domain death agonist

Vdcc	Signal transduction MAPK, Calcium,cAMP, cGMP - PKG signaling pathway	calcium channel, voltage-dependent, L type, alpha 1C subunit
Calm1	Signal transduction Ras , Rap1 , Calcium , Phosphatidylinositol,cAMP cGMP - PKG signaling pathway	calmodulin 1 (phosphorylase kinase, delta)
Caln	Signal transduction, MAPK, Wnt, VEGF Calcium, cGMP - PKG signaling pathway	protein phosphatase 3, catalytic subunit, alpha isozyme
CASP12	Folding, sorting and degradation	caspase 12
Casp3	Signal transduction MAPK, TNF signaling pathway	Caspase 3
Casp7	Signal transduction TNF signaling pathway	Caspase 7
Casp8	Signal transduction TNF signaling pathway	Caspase 8
Casp9	Signal transduction, VEGF,PI3K-Akt signaling pathway	Caspase 9
Cdk5	Axon guidance,Transferring phosphorus-containing groups	cyclin-dependent kinase 5
P25	Cdk activation	cyclin-dependent kinase 5 activator 1
CxI	Energy metabolism Oxidative phosphorylation	NADH dehydrogenase (ubiquinone) 1 subunit C2
CxII	Carbon metabolism, Carbohydrate metabolism Citrate cycle (TCA cycle) Oxidative phosphorylation	succinate dehydrogenase complex, subunit A, flavoprotein
CxIII	Energy metabolism Oxidative phosphorylation	ubiquinol-cytochrome c reductase, complex III subunit XI
CxvI	Energy metabolism Oxidative phosphorylation	cytochrome c oxidase subunit 6b
Cxv	Energy metabolism Oxidative phosphorylation	ATP synthase, H+ transporting, mitochondrial Fo complex, subunit d
Cycs	Energy metabolism Sulfur metabolism	cytochrome c, somatic

Perk	Genetic Information Processing<br>Folding, sorting and degradation	eukaryotic translation initiation factor 2-alpha kinase 3
Fadd	Signal transduction<br>TNF signaling pathway	FAS-associated death domain protein
Fas	Signal transduction<br>MAPK ,TNF signaling pathway	Fas cell surface death receptor<br>tumor necrosis factor receptor superfamily member 6
Appbp1	Ubiquitin-activating enzymes	amyloid beta precursor protein binding protein 1
Gapdh	Metabolism, Carbon metabolism<br>Biosynthesis of amino acids	glyceraldehyde-3-phosphate dehydrogenase
Gq	Signal transduction Rap1 ,Calcium, Sphingolipid,cGMP - PKG signaling pathway	guanine nucleotide binding protein (G protein), q polypeptide
Nmdar	Signal transduction<br>Ras ,Rap1,Calcium,cAMP signaling pathway	glutamate receptor, ionotropic, N-methyl D-aspartate 1
Gsk3b	Signal transduction ErbB,Wnt, Hedgehog, Hippo, PI3K-Akt signaling pathway	glycogen synthase kinase 3 beta
Ide	Metalloendopeptidase, insulsin	insulin-degrading enzyme
Il1b	Signal transduction<br>MAPK ,NF-kappa B, TNF signaling pathway	interleukin 1, beta
Ip3r	Signal transduction Calcium signaling pathway,Phosphatidylinositol signaling system cGMP - PKG signaling pathway	inositol 1,4,5-trisphosphate receptor, type 1
Ire1a	Genetic Information Processing<br>Folding, sorting and degradation	endoplasmic reticulum to nucleus signaling 1
Lpl	Lipid metabolism Glycerolipid metabolism	lipoprotein lipase
Lrp1	lipid metabolism,alpha-2-macroglobulin receptor	low density lipoprotein receptor-related protein 1
ERK/Mapk2	Signal transduction Ras,Rap1 , MAPK, ErbB ,TGF-beta VEGF ,TNF ,HIF-1 ,FoxO ,Phospholipase D ,Sphingolipid, cAMP , cGMP - PKG ,PI3K-Akt mTOR signaling pathway	mitogen-activated protein kinase 1
Mapt/Tau		microtubule-associated protein tau

	Signal transduction<br>MAPK signaling pathway	
Ncstn	Signal transduction<br>Notch signaling pathway	nicastrin
NEP/Mme	Immune system,	membrane metallo-endopeptidase, neprilysin
Nos1	Amino acid metabolism	nitric oxide synthase 1
Plcb1	Carbohydrate metabolism,Signal transduction	phospholipase C, beta 1 (phosphoinositide-specific)
Psen1	Signal transduction<br>Wnt, Notch,Neurotrophin signaling pathway	presenilin 1
Pen-2	Signal transduction<br>Notch signaling pathway	presenilin enhancer gamma secretase subunit
Ryr3	Signal transduction Calcium signaling<br>Oxytocin signaling pathway	ryanodine receptor 3
Snca	pre-Synaptic transmission, secondary	synuclein, alpha (non A4 component of amyloid precursor)
Tnf	Signal transduction<br>MAPK, TGF-beta, NF-kappa B , TNF ,Sphingolipid, mTOR, signaling pathway	tumor necrosis factor

Table S 2: Brief description of all the genes and their function involved in Alzheimer disease on set.
